# Explainable Deep Learning to Predict Kelp Geographical Origin from Volatile Organic Compound Analysis

**DOI:** 10.3390/foods14071269

**Published:** 2025-04-04

**Authors:** Xuming Kang, Zhijun Tan, Yanfang Zhao, Lin Yao, Xiaofeng Sheng, Yingying Guo

**Affiliations:** 1 Key Laboratory of Testing and Evaluation for Aquatic Product Safety and Quality, Ministry of Agriculture and Rural Affairs, Yellow Sea Fisheries Research Institute, Chinese Academy of Fishery Sciences, Qingdao 266071, China; kangxm@ysfri.ac.cn (X.K.); tanzj@ysfri.ac.cn (Z.T.); zhaoyf@ysfri.ac.cn (Y.Z.); yaolin@ysfri.ac.cn (L.Y.); shengxf@ysfri.ac.cn (X.S.); 2 State Key Laboratory of Mariculture Biobreeding and Sustainable Goods, Yellow Sea Fisheries Research Institute, Chinese Academy of Fishery Sciences, Qingdao 266071, China

**Keywords:** kelp, volatile organic compounds, explainable deep learning, SHAP, geographical origin

## Abstract

In addition to its flavor and nutritional value, the origin of kelp has become a crucial factor influencing consumer choices. Nevertheless, research on kelp’s origin traceability by volatile organic compound (VOC) analysis is lacking, and the application of deep learning in this field remains scarce due to its black-box nature. To address this gap, we attempted to identify the origin of kelp by analyzing its VOCs in conjunction with explainable deep learning. In this work, we identified 115 distinct VOCs in kelp samples using gas chromatography coupled with ion mobility spectroscopy (GC-IMS), of which 68 categories were discernible. Consequently, we developed a comprehensible one-dimensional convolutional neural network (1D-CNN) model that incorporated 107 VOCs exhibiting significant regional disparities (*p* < 0.05). The model successfully discerns the origin of kelp, achieving perfect metrics across accuracy (100%), precision (100%), recall (100%), F1 score (100%), and AUC (1.0). SHapley Additive exPlanations (SHAP) analysis highlighted the impact of features such as 1-Octen-3-ol-M, (+)-limonene, allyl sulfide-D, 1-hydroxy-2-propanone-D, and (*E*)-2-hexen-1-al-M on the model output. This research provides deeper insights into how critical product features correlate with specific geographic information, which in turn boosts consumer trust and promotes practical utilization in actual settings.

## 1. Introduction

Seaweed has been consumed for centuries in coastal regions around the world and serves as a staple in the daily diets of many cultures. Approximately 600 species of seaweed are utilized for human consumption, with brown seaweed being the most consumed type [1]. Among brown seaweeds, kelp is the most extensively produced variety and plays a significant role in East Asian diets. Several studies have indicated that seaweeds, including kelp, are highly nutritious foods with elevated levels of proteins, essential amino acids, minerals, fiber, and phenolic compounds, while being low in fat and possessing a favorable Na/K ratio [1,2,3,4,5,6]. However, from an alternative perspective, numerous incidents related to seaweed quality have emerged, including both chemical contamination and food safety risks such as the presence of inorganic arsenic compounds, excessive iodine levels, and harmful pathogens [7,8,9]. Additionally, the occurrence of dyed kelp has also been documented [10]. In recent years, individuals selecting kelp have increasingly prioritized flavor in addition to its nutritional value and potential hazards [11]. These factors are somewhat related to the origin of kelp. In this context, misleading labeling regarding kelp’s origin is unjust to consumers and may pose potential health risks. Additionally, certain products with protected geographical indications (PGIs), such as Rongcheng kelp, are susceptible to counterfeiting due to inadequate geographical origin traceability technology. Therefore, it is imperative to develop effective methods for determining the origin of kelp. This underscores the necessity of enhancing traceability techniques for kelp origin, with an emphasis on ensuring product quality, safety, and the protection of trademarks.

To safeguard the quality and safety of seaweed and maintain consumer confidence, various methods have been implemented to determine its origin. For instance, mineral elemental fingerprinting has been employed to ascertain the origin of red seaweed (*Neopyropia yezoensis*), green seaweed (*Ulva* spp.), and brown seaweed (*Fucus vesiculosus*) [12,13]. Additionally, near-infrared spectroscopy has been employed as an efficient tool for the swift identification of brown seaweed (*Sargassum fusiforme*) [14]. Stable isotope technology has also been applied to determine the origin of brown seaweed (*Undaria pinnatifida*) [15]. Nonetheless, there has been no prior documentation on the use of volatile organic compounds (VOCs) for tracing the origins of seaweed, especially kelp. Gas chromatography–ion mobility spectrometry (GC-IMS) is a rapid and widely used technique for analyzing VOCs in various food products. This technology provides several significant advantages, including a straightforward device setup, non-destructive analysis, environmental friendliness, continuous operation, and no need for sample pretreatment [16,17]. Furthermore, GC-IMS has proven effective in identifying the origin and species of various foods, including honey, soy sauce, and rice [17,18]. Accordingly, the analysis of kelp’s VOCs using GC-IMS is essential. It not only meets consumer demand for the flavor of kelp but also addresses the gap in utilizing this technology for tracing the origin of kelp.

As artificial intelligence continues to evolve, machine learning and deep learning techniques have become increasingly prevalent for data processing applications in food chemistry, especially in the field of food authenticity and traceability [19,20]. Convolutional neural networks (CNNs), widely used deep learning algorithms, have achieved significant success compared to traditional machine learning in image recognition tasks and classification problems [21,22]. To date, CNNs have been utilized for the traceability of various food products, demonstrating excellent model performance in items such as sea cucumbers, wolfberries, and semen ziziphi spinosae [22,23,24]. Nevertheless, the black-box nature of deep learning models, which refers to the characteristic that the internal workings or decision-making processes of a model are hidden or difficult for external observers to understand, often restricts their practical implementation in real-world scenarios [25]. The SHapley Additive exPlanations (SHAP) framework, which is a post hoc interpretability technique, has been employed to clarify the outputs generated by machine learning models [26], all while preserving the original performance of the trained models [27]. Additionally, SHAP provides a comprehensive understanding of how feature inputs influence model outputs, thereby enhancing the transparency of black-box models. Nevertheless, studies focusing on explainable machine learning or deep learning within the context of origin traceability remain exceedingly rare to this day [20,25,28].

This study aimed to develop an effective method for the accurate and rapid identification of kelp origins by integrating GC-IMS with explainable deep learning techniques. The primary research activities are outlined as follows: (1) identifying the VOCs present in kelp from different origins; (2) designing deep learning models using a one-dimensional convolutional neural network (1D-CNN) framework and assessing their feasibility; and (3) elucidating the contributions of key VOCs to the model decision-making process through SHAP explainability analysis at both global and local levels. This study seeks to significantly enhance the practical application of origin traceability techniques and predictive modeling by improving the speed and accuracy of kelp origin identification, as well as increasing the transparency and interpretability of the models employed.

## 2. Materials and Methods

### 2.1. Sampling

**Figure 1:**
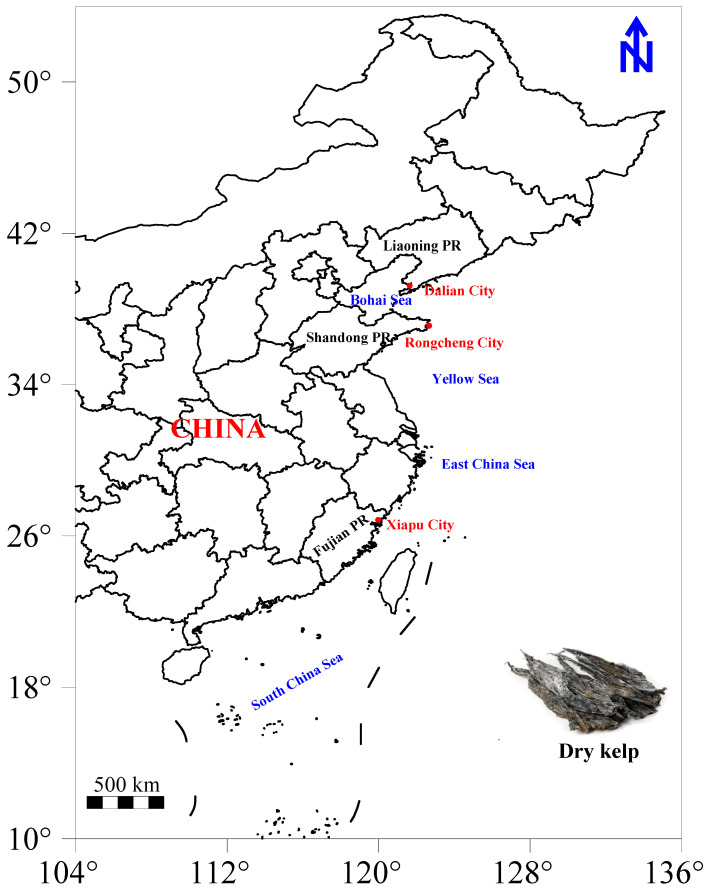
Spatial distribution of kelp samples collected from three provinces in China.

In 2023, it is estimated that China’s kelp production will surpass 1.78 million tons, with Fujian, Liaoning, and Shandong as the leading provinces for kelp aquaculture in the country [29]. These provinces have been designated as key monitoring regions. To ensure the authenticity of the samples, dried kelp (*Laminaria japonica*) was collected directly from different production enterprises in Dalian City (*n* = 30) in Liaoning Province, Rongcheng City (*n* = 30) in Shandong Province, and Xiapu City (*n* = 30) in Fujian Province in 2024 (Figure 1). For each batch of samples, 1 kg of dried kelp was carefully collected, placed in food-grade plastic bags, and sealed before transportation to the laboratory. All fresh kelp used for dried kelp production was sourced from coastal aquaculture farms near the production enterprises.

### 2.2. Sample Preparation and GC–IMS Analysis

The dried kelp was finely powdered using a grinding machine and then transferred into plastic bags. Subsequently, it was kept in a desiccator at ambient temperature until analysis. The VOCs in 90 kelp samples were analyzed using a GC-IMS system. This system consisted of a 490 GC unit from Agilent Technologies Inc. (Palo Alto, CA, USA) coupled with an IMS detector from Flavourspec^®^ (G.A.S., Dortmund, Germany). Approximately 1.5 g of the specimen was transferred into a 20 mL headspace vial and maintained at 60 °C with orbital shaking (500 rpm) for 15 min using an automated sampling system (CTC Analytics AG, Zwingen, Switzerland; CTC-PAL 3 model). Following this, a volume of 500 μL of gas was injected using a syringe that was heated to 85 °C. The gas chromatography process utilized an MXT-WAX column (15 m × 0.53 mm × 1.0 μm, Restek, Bellefonte, PA, USA) for separation, operating at a column temperature of 60 °C and with a total run time of 30 min. Nitrogen (N_2_, purity ≥ 99.999%) served as the carrier gas. Its flow rate was initially set at 2.0 mL/min for the first 2 min, was subsequently ramped up to 10.0 mL/min over the next 8 min, and then was linearly increased to 100 mL/min within 10 min, where it was held constant for an additional 10 min. The IMS conditions were as follows: the drift tube length was 98 mm, the temperature was set at 45 °C, and the drift gas was nitrogen (N_2_, purity ≥ 99.999%) with a flow rate of 150 mL/min. The ionization source was deuterium, and the ionization mode was positive ion. The retention index (RI) for each VOC was determined using n-ketones (C_4_–C_9_) as external standards, chosen for their minimal response in ion mobility spectrometry (IMS). The target VOCs were qualitatively analyzed by referencing the built-in GC RI database (NIST, 2020) and the IMS drift time (Dt) database in VOCal software (0.4.07). The VOC concentrations were quantitatively compared using peak volume signal intensity from the Laboratory Analytical Viewer (LAV) [30]. Meanwhile, fingerprints and differential profiles of volatile molecules in kelp were generated using the Reporter and Gallery plugins in VOCal software [31,32].

### 2.3. Modeling Procedure

The primary procedure for model building is outlined as follows: Firstly, a dataset was assembled from VOCs exhibiting marked regional discrepancies, organized into a 90-sample matrix. The matrix was structured with 90 rows indicative of discrete instances and 107 columns corresponding to individual attributes. The target matrix, configured as a 90 × 1 array, contained a column titled “origin label”, signifying the cities of Rongcheng, Dalian, and Xiapu, each designated with the unique identifiers 0, 1, and 2.

Secondly, the dataset (*n* = 90) was initially partitioned into training and testing subsets in a 7:3 ratio before any preprocessing operations were conducted. This approach was specifically adopted to avert data leakage during the model development phase, as suggested by Kapoor and Narayanan [33]. Following this division, the Standard Scaler was chosen for normalization. The *fit* () function was applied exclusively to the training set to calculate the scaling parameters, specifically the mean (μ) and standard deviation (σ), for each feature. Subsequently, the *transform* () function was utilized to standardize both the training and testing sets based on these parameters, ensuring that the data were normalized consistently across both subsets. For the training data, each feature value was transformed using the formula: X_train_normalized = (X_train − μ)/σ. For the test data, the same μ and σ values obtained from the training data were applied, with the formula being X_test_normalized = (X_test − μ)/σ. This process mitigates the adverse effects caused by variations in the concentrations of VOCs in kelp [34].

Thirdly, the preprocessed training subset (*n* = 63) was utilized to train the models, while their performance was assessed using the test subset (*n* = 27). A range of statistical indices were employed to comprehensively evaluate the models, including accuracy, precision, recall, and F1 score. These metrics are defined as follows:

(1) $$Accuracy=\frac{TP+TN}{TP+TN+FP+FN}$$

(2) $$Recall=\frac{TP}{TP+FN\;}$$

(3) $$Precision=\frac{TP}{TP+FP}$$

(4) $$F1\;score=\frac{2\times Precision\times Recall}{Precision+Recall}$$

In this context, TP refers to true positives, FP indicates false positives, TN signifies true negatives, and FN denotes false negatives. Furthermore, AUC represents the Area Under the Curve, which relates to the receiver operating characteristic (ROC) curve. This curve is created by plotting the FP rate against the TP rate [28]. Additionally, the computational complexity of the proposed model was evaluated based on the number of model parameters and floating-point operations (FLOPs).

### 2.4. One-Dimensional Convolutional Neural Network (1D-CNN)

**Figure 2:**
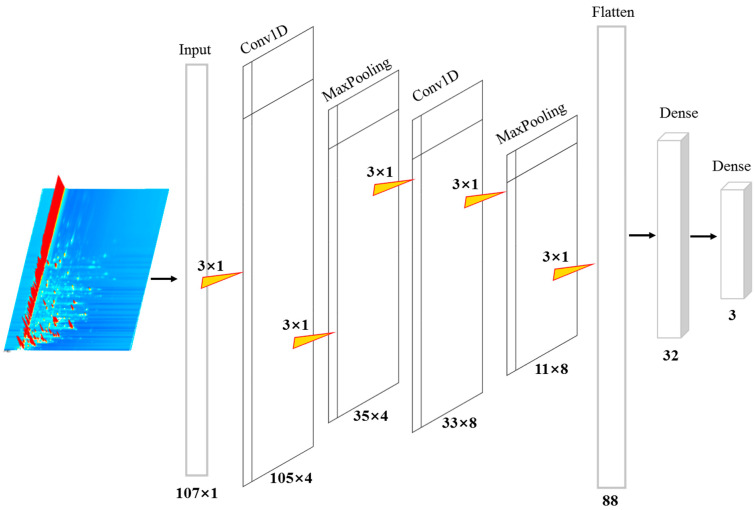
The architecture of the proposed 1D-CNN model for identification of kelp.

One-dimensional convolutional neural networks (1D-CNNs) share similarities with their two-dimensional counterparts (2D-CNNs) in that both utilize convolutional operations to extract features from input data. However, 1D-CNNs are particularly well suited for processing one-dimensional signals, such as spectral data. This advantage is especially pronounced when the available training data are limited or when the application is highly specialized, as 1D-CNNs can effectively capture relevant features and patterns within such data [21,22]. In this research, a 1D-CNN model was formulated to ascertain the geographical origin of kelp based on VOC data. The architecture of the 1D-CNN is depicted in Figure 2. More precisely, the 1D-CNN architecture features two convolutional layers, two max pooling layers, one flattened layer, and two dense layers. The input layer receives processed VOC data in a (107 × 1) format. During the convolution and max pooling operations, each convolutional kernel and max pooling filter retains dimensions of (3 × 1). The model employed a rectified linear unit (ReLU) activation function to introduce nonlinearity, thereby reducing computational complexity and alleviating the “dying ReLU” issue, which can impede the convergence of machine learning models during training [18,35]. The flattening layer converts the extracted and pooled VOC features into an 88-element one-dimensional vector, which is subsequently fed into a fully connected dense layer containing 32 neurons. At the network’s output, a softmax activation function is employed in the fully connected layer to assign sample labels by evaluating the predicted probabilities.

The Adam optimizer, an advanced variant of stochastic gradient descent [36], was utilized to train the 1D-CNN model for minimizing cross-entropy loss. The discrepancies between predicted and actual values were assessed using a categorical cross-entropy loss function [37].

### 2.5. Shapley Additive Explanations (SHAP)

We utilize a game theory-based SHAP method for the interpretative analysis of our deep learning model [26]. This method enables us to assess the impact of each feature on both the overall model and each predicted class. In this research, DeepExplainer, which computes SHAP values for deep learning models by leveraging the connections between SHAP and the DeepLIFT algorithm, was utilized. The contribution of each variable to the model’s output is evaluated by the SHAP algorithm, utilizing both the model itself and the input dataset, as detailed below [38]:

(5) $$\varphi_{i}=\sum_{S\subseteq F\backslash \left\{\left.i\right\}\right.}\frac{\left|S\right|!\left(|F|-|S|-1\right)!}{\left|F\right|!}\left[\left.f_{S\cup \left\{i\right\}}\left(x_{S\cup \left\{i\right\}}\right)-f_{S}(x_{S})\right]\right.\;$$

In this scenario, $\varphi_{i}$, *F,* and *S* denote the contribution of each individual feature, the complete set of features, and the subset of features that excludes the $i^{th}$ feature, respectively. Subsequently, two models are retrained: $f_{S\cup \{i\}}$, with the inclusion of the $i^{th}$ feature, and $f_{S},$ with its exclusion. The predictions from these models are compared using the equation $f_{S\cup \left\{i\right\}}(x_{S\cup \left\{i\right\}})-f_{S}(x_{S})$, where $x_{s}$ represents the values of the input features within the set S.

### 2.6. Computing Implementation

All the computational steps outlined above, which cover the preprocessing of raw data, model development, and interpretation, were carried out with Python 3.8 and PyCharm Community Edition 2021.3.1, along with the scikit-learn 0.24.2 (available at GitHub: https://github.com/scikit-learn (accessed on 10 October 2024)), TensorFlow 2.2.0 (available at GitHub: https://github.com/tensorflow/tensorflow (accessed on 11 October 2024)), keras-flops 0.1.0 (available at GitHub: https://github.com/tokusumi/keras-flops (accessed on 24 March 2025)), and SHAP 0.41.0 libraries (available at GitHub: https://github.com/slundberg/shap (accessed on 15 October 2024)).

## 3. Results and Discussion

### 3.1. Identification of VOCs in Kelp by GC-IMS

**Figure 3:**
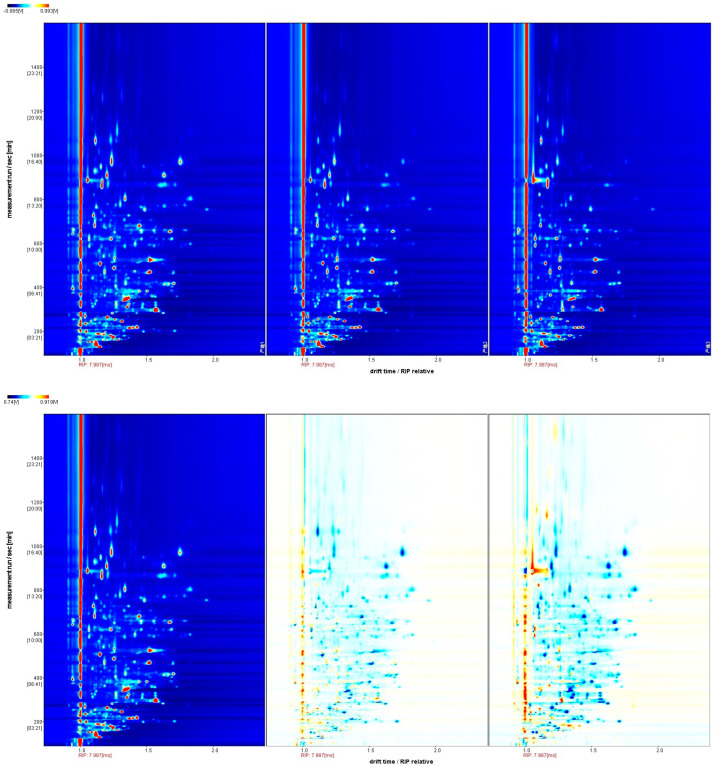
Gas chromatography–ion mobility spectrometry spectra of VOCs. The left panel: Rongcheng kelp; middle panel: Dalian kelp; right panel: Xaipu kelp. Note: The background of the figure is blue, and the color indicates the peak intensity of the VOCs, with deeper colors from blue to red indicating higher peak intensity. A comparison is given with reference sample spectra (Rongcheng kelp), with red or blue indicating VOC concentrations higher or lower than the reference, while white indicates the same concentration as the reference.

The VOCs present in kelp sourced from three cities were analyzed using GC-IMS. The abscissa represents the relative ion drift time (Dt), whereas retention time is plotted along the ordinate. The crimson reference marker positioned at x = 1.0 delineates reactive ion peak (RIP) baselines, while a chromatic gradient correlates with VOC signal magnitudes through an intensity-dependent colorimetric scale (blue < red progression) [39]. Our analysis demonstrates predominant spectral features are confined to retention time (100–1100 s) and drift time (Dt 1.0–1.8 ms) operational windows, while co-detected analyte signatures exhibit temporal synchronization across both separation dimensions (Figure 3, upper panel). This observation underscores the variations in properties including volatility, polarity, molecular weight, and charge state of the VOCs [40].

To achieve a more comprehensive grasp of the regional disparities in VOCs among kelp, the spectral data of Rocheng kelp were utilized as a benchmark for comparison with other samples’ spectra. When the VOCs in the samples align with those of the reference, the resulting background after subtraction is white; in contrast, red denotes a VOC concentration surpassing the reference level, while blue indicates a reduced concentration, with darker hues signifying a more pronounced difference. Our analysis clearly illustrates that the VOC profiles vary among the three cities (Figure 3, bottom panel).

**Figure 4:**
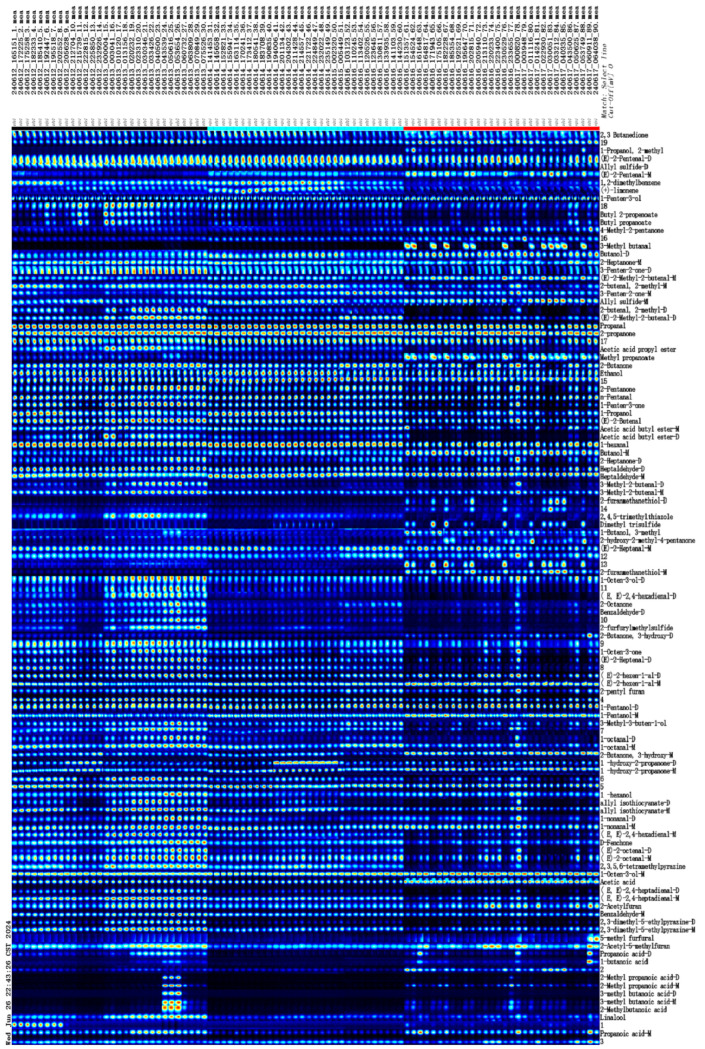
Gallery plot of VOCs in kelp from different origins. M: monomer; D: dimer. Each row represents one sample and each column represents a VOC. The upper 30 samples are from Rongcheng, the middle 30 samples are from Dalian, and the bottom 30 samples are from Xiapu.

GC-IMS analysis reveals significant diversity in the VOCs present in kelp, with 115 distinct VOCs identified. Each compound exhibits detection signals across all samples, albeit at varying concentration levels (Figure 4). Among the 115 detected VOCs, 96 were identified. These identified VOCs can be categorized into 68 distinct species, which include 19 aldehydes, 14 ketones, 12 alcohols, 6 esters, 6 acids, 3 furans, 2 pyrazines, 2 ethers, and 4 others (Table 1). However, because of the insufficient data in the database, 19 VOCs were without qualitative results, as shown by their numerical labels. It is worth noting that several VOCs with higher levels, such as 3-methylbutanoic acid, 2,3-dimethyl-5-ethylpyrazine, (*E,E*)-2,4-heptadienal, 1-octen-3-ol, allyl sulfide, allyl isothiocyanate, and 1-hydroxy-2-propanone, generated monomer and dimer compounds that had similar retention times but different drift times (Figure 4) [41]. The majority of VOCs identified in kelp align with those reported in prior research [39,42,43]. The identified allyl isothiocyanate in kelp is primarily generated from precursor glucosinolates through enzymatic hydrolysis [44]. Aldehydes are significant secondary products of lipid oxidation, originating from the decomposition of hydroperoxides. In addition to this, certain aldehydes can also be generated through the degradation of amino acids induced by the Maillard reaction [31]. Alcohols identified in seaweed are predominantly derived from the peroxidation of unsaturated fatty acids, while ketones are generated through the oxidation or breakdown of both unsaturated fatty acids and amino acids [11]. Esters are synthesized by esterases produced by *Monascus* spp., facilitating the esterification of acids and alcohols [45].

**Figure 5:**
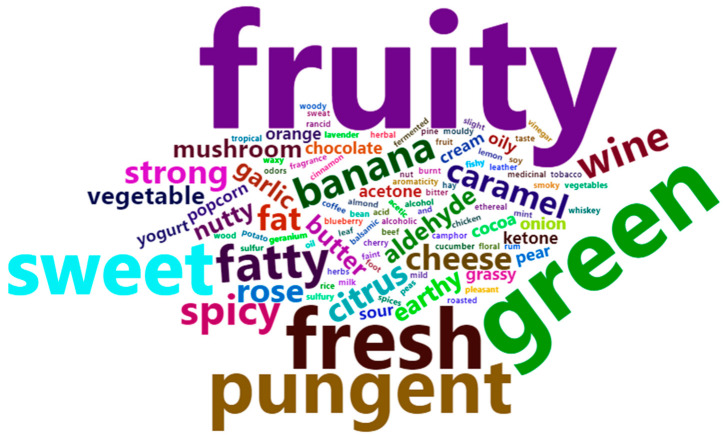
Word clouds of odor descriptors for VOCs in kelp.

Flavor, a complex perceptual factor encompassing both odor and taste, is crucial for food acceptance. As a marine-derived food product, kelp exhibits a unique oceanic flavor shaped by its growth environment [39]. Although the VOCs detected in kelp vary across different studies, it is evident that these compounds significantly influence its flavor [43]. The odor descriptors assigned to each VOC detected via GC-IMS in this research are documented within Table 1 and are visually represented as word clouds in Figure 5. The word clouds visualize the frequency of descriptors through increasing size and font weight [46]. Overall, the odor of the detected VOCs in kelp is characterized by a dominant perception of “fruity” and “green”, along with moderate perceptions of “sweet”, “fresh”, and “pungent”. For instance, “fruity” and “green” were supplemented with a variety of descriptors, including those typically associated with acids, aldehydes, alcohols, esters, and ketones (e.g., cheese, banana, citrus, peas), as well as unexpected ones (e.g., foot sweat, sulfury, garlic). Similarly, “sweet”, “fresh”, and “pungent” were augmented by a range of descriptors, including those expected for aldehydes, alcohols, furan, and esters (e.g., citrus, wine, caramel), as well as unexpected ones (e.g., tobacco, acetone, grassy). According to Wei et al. [39], sensory attributes, including “green”, “fatty”, and “cucumber”, are identified as significant contributors to the development of fishy odors in kelp. This diversity and non-uniformity in odor descriptors often provide more references for consumers when selecting different origins of kelp.

Given that Figure 4 fails to clearly depict the variations in VOCs among kelp from different origins, the concentration intensity signals and standard deviations of the VOCs are displayed in Table 1. The one-way analysis of variance indicates that, with the exception of the volatile substances 3-methylbutanoic acid-M, 3-methylbutanoic acid-D, 2-methylbutanoic acid, 2-methylpropanoic acid-D, (*E*)-2-heptenal-M, 2,3-butanedione, and two unidentified VOCs denoted as numbers 3 and 12, the remaining 107 VOCs exhibit significant regional differences (*p* < 0.05). Previous research has identified factors including species, collection season, geographical origin, and pretreatment procedures as influencing the VOCs of seaweed samples [47]. Consequently, we hypothesize that the 107 VOCs, which exhibit significant regional differences, may serve as effective markers for identifying the origin of kelp. Chu et al. [25] adopted an inverse methodology, initially modeling the data via a data-driven approach and subsequently examining the learned weights to pinpoint features with substantial significance or potential for classification. Therefore, in the next section, we implemented an explainable, data-driven 1D-CNN model to validate our hypothesis.

### 3.2. Model Performance of 1D-CNN

**Figure 6:**
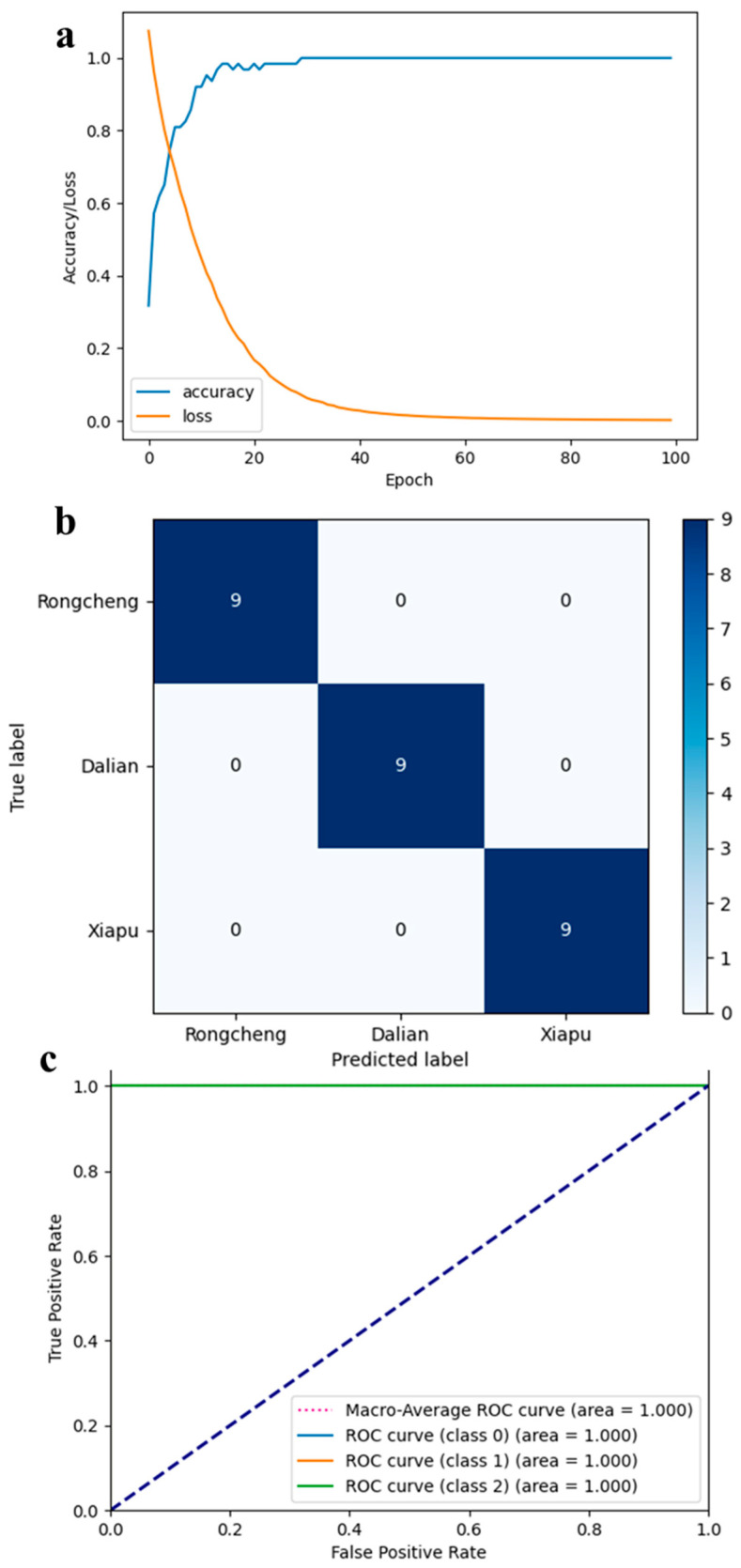
Performance of 1D-CNN model: (**a**) accuracy and cross-entropy loss curves during training; (**b**) receiver operating characteristic (ROC) curves of the model for each class; (**c**) confusion matrix of test set for each class.

The 1D-CNN model was assessed using the test set assigned in the preceding section. The evaluation results revealed that the model achieved perfect performance, with discrimination accuracy, recall, and F1 score all reaching 100%. To further assess the 1D-CNN model’s performance and its ability to distinguish between geographical origins, we documented and visualized the training accuracy, cross-entropy loss, AUC, and confusion matrix for each origin in Figure 6. As depicted in Figure 6a, the training set’s accuracy improved with the increase in epochs, while the cross-entropy loss declined, demonstrating that the 1D-CNN model rapidly converged within 100 epochs. As illustrated in Figure 6b, the confusion matrix reveals that all 27 test set samples were accurately classified. Additionally, the diagnostic ability of the 1D-CNN model was evaluated using the ROC curve presented in Figure 6c, with the AUC for samples from all three cities reaching 1.00. Furthermore, the proposed 1D-CNN model exhibits lower computational complexity, with FLOPs totaling only 16.1 K and model parameters comprising just 3.1 K, making it easy to reproduce elsewhere. Compared to the predictive accuracy of 95.8% achieved by earlier studies using fatty acid fingerprinting technology to trace the origin of kelp, our method achieves an accuracy of 100% [48]. Consequently, the 1D-CNN model developed in this study is highly effective in identifying the origin of kelp. In summary, the model presents remarkable precision, swift convergence, and robust generalization in determining the geographical origins of kelp from major cities in China.

### 3.3. Interpretation of 1D-CNN Model with SHAP Values

Evaluating the practicality of deep learning models hinges on transparency and interpretability. Yet, models employing complex nonlinear algorithms tend to be convoluted, potentially entailing intricate interactions among numerous factors or features, which complicates users’ grasp of the output rationale. Typically, post hoc explanations are utilized for sophisticated machine learning models [49]. Consequently, SHAP is utilized to discern the core of specific predictions, grasp the interplay between variables and model outcomes, and shed light on particular instances. By evaluating the mean incremental contribution of features through SHAP values, this method deepens our grasp of predictions within specific contexts. It also furnishes important insights into both singular cases and overarching trends at both global and local levels [50]. The utilization of SHAP interpretation has been crucial in building trust within food traceability systems, such as those for lotus and oysters [28,51]. Overall, this method is crucial for evaluating models and enhancing their practicality.

#### 3.3.1. Global Feature Interpretation

**Figure 7:**
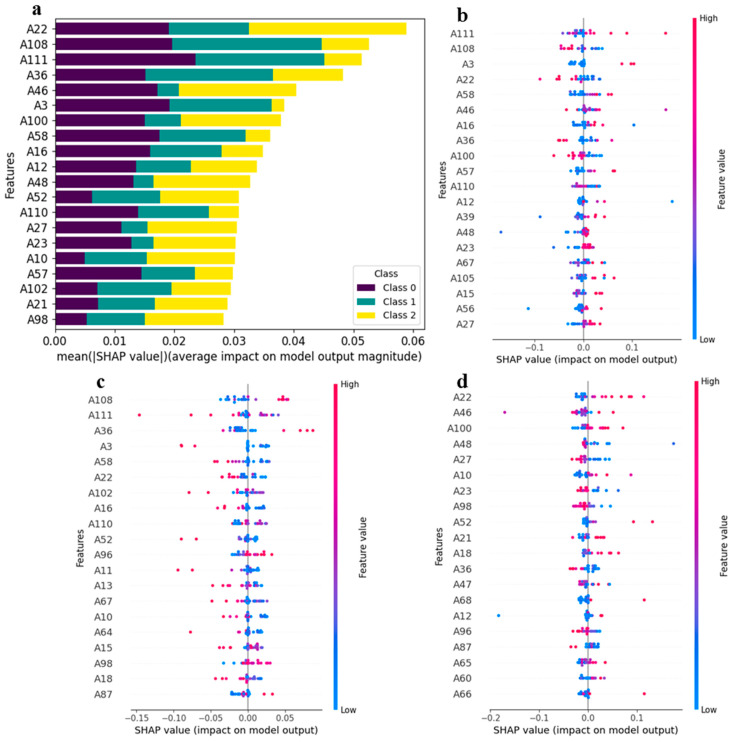
Global interpretation of the 1D-CNN model based on SHAP values: (**a**) the feature importance for the 1D-CNN model; (**b**–**d**) the feature contributions for predicting samples from Rongcheng, Dalian, and Xiapu, respectively. The color bar, ranging from blue to red, indicates the magnitude of feature values from low to high. Additionally, the position of the points on the horizontal axis denotes the positive or negative association between the features and target variables (refer to Table 1 for feature names).

The SHAP toolkit was applied to the trained 1D-CNN model, generating a matrix of SHAP values. To pinpoint the most influential features for the 1D-CNN model, we computed the mean absolute SHAP values for each feature and generated a stacked bar plot, with different geographical origins represented by distinct colors (Figure 7a). In this plot, the top 20 features were ranked according to the absolute sum of their impacts on the model. Notably, the features A22 (1-Octen-3-ol-M), A108 ((+)-limonene), A111 (allyl sulfide-D), A36 (1-hydroxy-2-propanone-D), and A46 ((*E*)-2-hexen-1-al-M) were identified as the top five features influencing the model output. Among these, feature A22 (1-Octen-3-ol-M) exhibited a dominant position, accounting for nearly 50% of the variance. This finding suggests that the concentration of A22 (1-Octen-3-ol-M) is the most crucial factor in the 1D-CNN model’s classification of kelp origin, particularly for Xiapu kelp.

Since SHAP values are derived from an individualized model interpretation approach, the contributions of features for each sample can be obtained from this interpretation. Figure 7 provides detailed plots illustrating the feature contributions to the model output for different origin classifications, with color representing the feature values. In the predictions for Rongcheng samples, A111 (allyl sulfide-D), A108 ((+)-limonene), A3 (1), A22 (1-octen-3-ol-M), and A58 (11) emerged as the top five features with the highest contributions. It is evident that samples with high feature values of A111 (allyl sulfide-D), A3 (1), and A58 (11) positively impacted the model, whereas contributions from features such as A56 (2-octanone) and A27 ((*E, E*)-2,4-hexadienal-M) were not significant (Figure 7b). For the predictions of Dalian samples, A108 ((+)-limonene), A111 (allyl sulfide-D), A36 (1-hydroxy-2-propanone-D), A3 (1), and A58 (11) again ranked among the top five features with the highest contributions. Here, samples with high feature values of A111 (allyl sulfide-D), A3 (1), and A58 (11) negatively impacted the model, while contributions from other features, such as A18 (2-acetylfuran) and A87 (methyl propanoate), were also minimal (Figure 7c). In the case of predictions for Xiapu samples, the top five features with the highest contributions were A22 (1-octen-3-ol-M), A46 ((*E*)-2-hexen-1-al-M), A100 (butanol-D), A48 (8), and A27 ((*E, E*)-2,4-hexadienal-M). Notably, samples with high feature values of A22 (1-octen-3-ol-M), A46 ((*E*)-2-hexen-1-al-M), and A100 (butanol-D) positively impacted the model, while high feature values of A27 ((*E, E*)-2,4-hexadienal-M) had a negative impact (Figure 7d). The findings suggest that the discriminative power of the 1D-CNN model for Rongcheng, Dalian, and Xiapu is affected by samples with high or low feature values.

#### 3.3.2. Local Feature Interpretation

**Figure 8:**
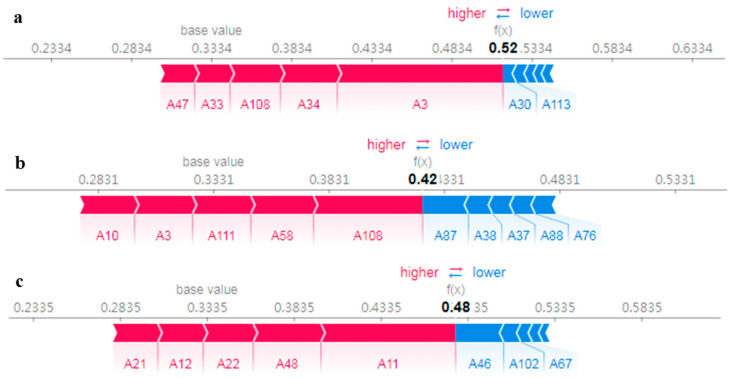
Local interpretation based on SHAP values for instances selected from (**a**) Rongcheng, (**b**) Dalian, and (**c**) Xiapu. Red feature attributions push the prediction higher than the base value (i.e., the mean model prediction over the training dataset), while blue feature attributions push the prediction lower. The size of the bars depicts importance (refer to Table 1 for feature names).

Interpretation of individual instances was executed with a SHAP force diagram (Figure 8), which displays samples drawn from the actual estimation outcomes for each city. In the graph, the baseline value signifies the mean model estimation results derived from the training dataset. When the model’s output lies to the right of the baseline value, the sample is categorized as belonging to the selected city; in contrast, if it lies to the left, the sample is not categorized as belonging to that city. This study focuses exclusively on the top five features that significantly influence the model output. The outputs of the model for three instances are shown to be positioned to the right of the base value, implying that these samples pertain to the selected city. However, the significance of each feature varies across different instances. In the case of Rongcheng, the base value is 0.3334, while the selected samples display a relatively higher prediction of 0.52 compared to the actual label of 0. This illustrates how certain values of A30 (allyl isothiocyanate-M) and A113 (1-propanol, 2-methyl) are associated with negative SHAP values, which consequently diminish the likelihood of samples belonging to Rongcheng. In contrast, the values of A3 (1), A34 (6), A108 ((+)-limonene), A47 ((*E*)-2-hexen-1-al-D), and A33 (5) are linked to positive SHAP values, thus increasing the probability that samples belong to Rongcheng (Figure 8a). For the Dalian cases, the values of A108 ((+)-limonene), A58 (11), A111 (allyl sulfide-D), A3 (1), and A10 (2) are associated with positive SHAP values (Figure 8b), while A87 (methyl propanoate), A38 (1-octanal-M), A37 (2-butanone, 3-hydroxy-M), A88 (acetic acid propyl ester), and A76 (1-hexanal) exert a negative influence on the Dalian cases (Figure 8b). The selected samples have a notably higher predicted value of 0.48 for Xiapu, with A11 (1-butanoic acid), A48 (8), A22 (1-octen-3-ol-M), A12 (propanoic acid-D), and A21 (acetic acid) related to positive SHAP values (Figure 8c). In summary, the tendency observed in the interpretation of local features is in parallel with that of global features.

## 4. Conclusions

This research revealed that combining GC-IMS with explainable deep learning offers a viable and efficient method for determining the geographical origin of kelp in China. A total of 115 VOCs were identified, with 107 showing marked regional variations. Moreover, the findings suggest that the proposed 1D-CNN model achieves an accuracy rate of 100%, good convergence, and excellent generalization ability when classifying kelp from three different cities. Additionally, the SHAPs for the 1D-CNN model highlighted the contribution of 1-octen-3-ol-M, (+)-limonene, allyl sulfide-D, 1-hydroxy-2-propanone-D, and (*E*)-2-hexen-1-al-M in the decision-making process of the model. The research findings reveal causal linkages among various features, thereby bolstering user confidence and guaranteeing the dependable practical implementation of the models. This pioneering work goes beyond the conventional development and application of deep learning, significantly promoting interpretability in the context of seaweed origin identification. In the final analysis, this study will aid in combating food fraud and safeguarding food security. Despite the successful accomplishment of the research goals, subsequent studies will also concentrate on more in-depth qualitative and quantitative investigations of unidentified VOCs. Furthermore, other technologies such as Fourier Transform Infrared Spectroscopy (FT-IR) and Ultraviolet–Visible Spectrophotometry (UV-Vis) should be considered in future research for qualitative and quantitative investigations of VOCs.

## Figures and Tables

**Table 1:** GC-IMS integration parameters of volatile compounds in kelp from different cities.

Features	Compound	RI	Rt [sec]	Dt [a.u.]	Signal Intensities			*p* Value	Odor Description
					**Rongcheng**	**Dalian**	**Xiapu**		
	**Acids**								
A6	3-Methyl butanoic acid-M	1704.9	1517.861	1.23403	1755.2 ± 3254.1 ^a^	707.6 ± 102.0 ^a^	1758.4 ± 936.9 ^a^	0.062	sour, foot sweat, cheese
A7	3-Methyl butanoic acid-D	1705.2	1519.095	1.49786	1254.2 ± 1993.5 ^a^	1088.8 ± 191.3 ^a^	1232.0 ± 210.2 ^a^	0.837	
A5	2-Methylbutanoic acid	1704.2	1515.632	1.20697	917.1 ± 1550.6 ^a^	470.9 ± 96.8 ^a^	880.5 ± 445.2 ^a^	0.127	pungent and spicy cheese, fruity
A8	2-Methyl propanoic acid-M	1578.1	1136.913	1.15979	1705.9 ± 2698.7 ^ab^	864.3 ± 177.4 ^b^	2534.7 ± 1173.2 ^a^	0.001	yogurt, rancid cream
A9	2-Methyl propanoic acid-D	1579	1139.117	1.38345	1026.4 ± 1904.3 ^a^	615.5 ± 110.0 ^a^	815.3 ± 369.7 ^a^	0.370	
A21	Acetic acid	1470.1	888.662	1.05409	5158.9 ± 378.3 ^b^	4404.3 ± 402.2 ^c^	8421.3 ± 728.6 ^a^	<0.001	spicy
A2	Propanoic acid-M	1548.1	1061.727	1.1132	3851.4 ± 1081.0 ^a^	1395.8 ± 210.0 ^b^	3565.2 ± 1331.5 ^a^	<0.001	yogurt, vinegar
A12	Propanoic acid-D	1548.1	1061.727	1.27443	698.1 ± 401.3 ^a^	248.5 ± 36.8 ^b^	548.7 ± 465.0 ^a^	<0.001	
A11	1-Butanoic acid	1647.1	1330.54	1.17401	442.5 ± 247.0 ^b^	390.6 ± 71.5 ^b^	739.5 ± 483.0 ^a^	<0.001	strong acetic acid, cheese, butter, fruity
	**Pyrazines**								
A16	2,3-Dimethyl-5-ethylpyrazine-D	1507.9	968.664	1.75452	3989.6 ± 1749.1 ^a^	1442.4 ± 280.9 ^b^	994.2 ± 421.7 ^b^	<0.001	burnt popcorn, roasted cocoa
A15	2,3-Dimethyl-5-ethylpyrazine-M	1510.5	974.378	1.23681	5998.3 ± 825.3 ^a^	4251.7 ± 379.3 ^b^	3192.7 ± 922.7 ^c^	<0.001	
A23	2,3,5,6-Tetramethylpyrazine	1460.7	869.986	1.2092	1148.4 ± 203.0 ^a^	1002.0 ± 167.5 ^b^	374.9 ± 122.7 ^c^	<0.001	beef, fermented soy
	**Aldehydes**								
A19	(*E, E*)-2,4-Heptadienal-M	1480.8	910.703	1.20277	4181.0 ± 489.5 ^a^	3231.2 ± 228.4 ^b^	1916.9 ± 776.0 ^c^	<0.001	fatty, oily, aldehyde, vegetable, cinnamon
A20	(*E, E*)-2,4-Heptadienal-D	1480.4	909.887	1.63091	3028.0 ± 1261.9 ^a^	1200.4 ± 236.6 ^b^	674.4 ± 375.4 ^c^	<0.001	
A17	Benzaldehyde-M	1500.4	952.337	1.15978	1759.6 ± 206.0 ^a^	1555.0 ± 118.3 ^b^	1395.3 ± 236.2 ^c^	<0.001	bitter almond, cherry, nutty
A55	Benzaldehyde-D	1498.5	948.255	1.47865	503.9 ± 184.6 ^a^	306.3 ± 35.2 ^b^	276.6 ± 86.8 ^b^	<0.001	
A24	(*E*)-2-Octenal-M	1427.8	807.027	1.33713	2655.6 ± 374.3 ^a^	2123.9 ± 236.8 ^b^	1372.5 ± 726.6 ^c^	<0.001	fresh cucumber, fatty, green herbal, banana, green leaf
A25	(E)-2-Octenal-D	1427.4	806.211	1.82976	1602.5 ± 668.1 ^a^	845.8 ± 247.3 ^b^	526.1 ± 459.1 ^c^	<0.001	
A28	1-Nonanal-M	1397	752.332	1.48402	1931.3 ± 237.0 ^a^	1701.6 ± 271.8 ^b^	1782.3 ± 424.6 ^ab^	0.023	rose, citrus, strong oily
A29	1-Nonanal-D	1398.9	755.597	1.95516	741.1 ± 298.0 ^a^	454.5 ± 114.7 ^b^	463.1 ± 242.6 ^b^	<0.001	
A14	5-Methyl furfural	1557.9	1085.791	1.13658	206.6 ± 54.4 ^b^	170.7 ± 26.8 ^b^	306.7 ± 177.0 ^a^	<0.001	spices, caramel wood
A27	(*E, E*)-2,4-Hexadienal-M	1405.7	767.443	1.12077	1291.0 ± 218.0 ^a^	902.4 ± 115.6 ^b^	498.5 ± 199.8 ^c^	<0.001	sweet, green, floral, citrus
A57	(*E, E*)-2,4-Hexadienal-D	1406.5	768.733	1.45577	595.3 ± 302.4 ^a^	216.9 ± 61.0 ^b^	113.6 ± 70.8 ^c^	<0.001	
A63	(*E*)-2-Heptenal-M	1336.2	654.959	1.25583	744.0 ± 124.3 ^a^	768.4 ± 140.0 ^a^	720.2 ± 177.3 ^a^	0.458	spicy, green vegetables, fresh, fatty
A49	(*E*)-2-Heptenal-D	1336.2	654.959	1.67623	3101.0 ± 865.8 ^a^	1952.0 ± 390.4 ^b^	1001.2 ± 761.1 ^c^	<0.001	
A72	Heptaldehyde-M	1191.2	419.599	1.34079	1518.4 ± 210.9 ^b^	1708.4 ± 137.0 ^a^	1559.6 ± 153.1 ^b^	<0.001	fresh, aldehyde, fatty, green herbs, wine, fruity
A73	Heptaldehyde-D	1191.2	419.599	1.70179	2101.4 ± 269.1 ^a^	1725.6 ± 187.8 ^b^	1276.9 ± 539.7 ^c^	<0.001	
A76	1-Hexanal	1094.8	297.552	1.56891	9942.1 ± 532.5 ^a^	10087.0 ± 215.3 ^a^	6551.3 ± 2462.1 ^b^	<0.001	fresh, green, fat, fruity
A82	n-Pentanal	994.8	219.957	1.42916	3180.9 ± 171.2 ^a^	3016.9 ± 121.4 ^a^	2361.7 ± 528.9 ^b^	<0.001	green grassy, faint banana, pungent
A101	3-Methyl butanal	925.3	183.139	1.4087	63.9 ± 24.0 ^b^	71.6 ± 12.0 ^b^	1043.7 ± 991.7 ^a^	<0.001	chocolate, fat
A97	(*E*)-2-Methyl-2-butenal-M	1108.9	312.596	1.09283	581.3 ± 103.0 ^c^	678.0 ± 59.0 ^b^	919.1 ± 177.0 ^a^	<0.001	
A92	(*E*)-2-Methyl-2-butenal-D	1107.8	311.409	1.34962	3436.3 ± 1152.5 ^a^	2576.4 ± 718.5 ^b^	1381.0 ± 511.7 ^c^	<0.001	
A110	(*E*)-2-Pentenal-M	1143.2	353.373	1.1042	243.0 ± 58.7 ^c^	433.2 ± 57.0 ^b^	575.4 ± 151.6 ^a^	<0.001	potato, peas
A112	(*E*)-2-Pentenal-D	1142.2	352.186	1.36666	2885.9 ± 284.6 ^a^	2944.2 ± 215.2 ^a^	2250.4 ± 557.5 ^b^	<0.001	
A46	(*E*)-2-Hexen-1-al-M	1224.9	470.305	1.18185	2112.4 ± 282.8 ^c^	2444.0 ± 112.4 ^b^	2813.3 ± 334.5 ^a^	<0.001	green, banana, fat
A47	(*E*)-2-Hexen-1-al-D	1226.6	473.163	1.52571	5560.2 ± 1109.4 ^a^	3992.7 ± 766.5 ^b^	4026.1 ± 1971.6 ^b^	<0.001	
A70	3-Methyl-2-butenal-M	1207.6	443.624	1.09281	688.1 ± 123.6 ^a^	536.8 ± 61.3 ^c^	604.1 ± 124.9 ^b^	<0.001	fruity
A71	3-Methyl-2-butenal-D	1208.5	445.053	1.36405	628.5 ± 303.6 ^a^	250.7 ± 53.7 ^b^	253.5 ± 204.1 ^b^	<0.001	
A91	Propanal	800.4	131.726	1.1456	5194.1 ± 136.1 ^a^	5008.8 ± 217.8 ^a^	4134.7 ± 801.0 ^b^	<0.001	pungent, green grassy
A79	(*E*)-2-Butenal	1057.6	265.853	1.20228	4939.1 ± 931.4 ^a^	3604.7 ± 280.9 ^b^	2657.7 ± 818.8 ^c^	<0.001	
A38	1-Octanal-M	1295.2	596.509	1.40975	1473.4 ± 229.0 ^a^	1288.3 ± 199.1 ^b^	899.4 ± 278.4 ^c^	<0.001	aldehyde, waxy, citrus, orange, fruity, fatty
A39	1-Octanal-D	1297.6	599.763	1.82402	697.4 ± 316.2 ^a^	384.0 ± 118.7 ^b^	289.6 ± 167.4 ^b^	<0.001	
A93	2-Butenal, 2-methyl-D	1120.2	325.542	1.3698	1038.4 ± 475.9 ^a^	611.4 ± 132.7 ^b^	184.7 ± 163.3 ^c^	<0.001	
A96	2-Butenal, 2-methyl-M	1122.3	327.897	1.11449	463.3 ± 100.0 ^a^	514.6 ± 112.2 ^a^	275.9 ± 94.9 ^b^	<0.001	
	**Alcohols**								
A22	1-Octen-3-ol-M	1457	862.538	1.16157	4006.9 ± 129.7 ^b^	4000.7 ± 264.4 ^b^	4565.8 ± 390.3 ^a^	<0.001	mushroom, lavender, rose, hay
A59	1-Octen-3-ol-D	1457	862.538	1.60583	1842.4 ± 383.1 ^a^	1265.9 ± 231.0 ^b^	1257.5 ± 474.7 ^b^	<0.001	
A4	Linalool	1549.8	1065.809	1.22785	1905.6 ± 582.5 ^a^	1153.8 ± 176.9 ^b^	1182.1 ± 286.8 ^b^	<0.001	citrus, rose, woody, blueberry
A80	1-Propanol	1046.1	256.775	1.25872	1757.2 ± 330.5 ^a^	1844.2 ± 187.8 ^a^	1105.3 ± 409.1 ^b^	<0.001	alcohol, pungent
A107	1-Penten-3-ol	1166.9	384.649	0.93944	2660.7 ± 253.7 ^c^	3093.6 ± 213.5 ^b^	3302.9 ± 204.0 ^a^	<0.001	ethereal, green, tropical fruity
A42	1-Pentanol-M	1259	527.955	1.25583	2333.4 ± 400.3 ^c^	2918.4 ± 261.3 ^b^	3308.2 ± 547.3 ^a^	<0.001	balsamic
A43	1-Pentanol-D	1258.3	526.526	1.5216	4883.0 ± 321.2 ^a^	4574.2 ± 552.3 ^ab^	4276.7 ± 860.1 ^b^	0.001	
A85	Ethanol	940.9	190.799	1.13695	3377.6 ± 347.3 ^b^	3790.7 ± 595.2 ^a^	2907.5 ± 625.4 ^c^	<0.001	aromaticity
A113	1-Propanol, 2-methyl	1102.8	305.806	1.17346	43.4 ± 6.17 ^b^	58.3 ± 17.1 ^b^	195.2 ± 114.6 ^a^	<0.001	fresh, alcoholic, leather
A75	Butanol-M	1153.4	366.495	1.18517	626.2 ± 103.7 ^c^	936.1 ± 132.4 ^b^	1324.6 ± 202.1 ^a^	<0.001	wine
A100	Butanol-D	1153	366.058	1.39075	431.5 ± 84.6 ^c^	584.5 ± 108.4 ^b^	690.2 ± 101.0 ^a^	<0.001	
A60	2-Furanmethanethiol-M	1436.6	823.458	1.10216	340.1 ± 41.1 ^c^	583.2 ± 129.5 ^b^	1293.7 ± 510.0 ^a^	<0.001	sulfury, coffee, fat, smoky
A69	2-Furanmethanethiol-D	1436.1	822.467	1.35824	111.4 ± 14.5 ^b^	126.0 ± 16.2 ^b^	346.4 ± 244.0 ^a^	<0.001	
A32	1-Hexanol	1369.8	707.132	1.32947	613.9 ± 162.7 ^a^	466.2 ± 80.7 ^b^	548.7 ± 138.6 ^a^	<0.001	fresh, fruity, wine, sweet, green
A41	3-Methyl-3-buten-1-ol	1267.3	542.825	1.17532	945.4 ± 190.5 ^a^	847.2 ± 198.1 ^a^	624.4 ± 212.7 ^b^	<0.001	sweet, fruity
A65	1-Butanol, 3-methyl	1213.6	452.748	1.24885	379.4 ± 116.5 ^b^	373.0 ± 79.8 ^b^	606.4 ± 163.8 ^a^	<0.001	whiskey, banana, fruity
	**Ether**								
A53	2-Furfurylmethylsulfide	1529.9	1018.461	1.14007	669.3 ± 369.7 ^a^	374.0 ± 109.5 ^b^	256.2 ± 74.5 ^c^	<0.001	pungent, onion, garlic
A94	Allyl sulfide-M	1135.3	343.522	1.12292	534.2 ± 102.8 ^c^	657.0 ± 142.8 ^b^	727.3 ± 155.4 ^a^	<0.001	garlic
A111	Allyl sulfide-D	1136.9	345.475	1.32433	3624.2 ± 608.1 ^a^	2616.3 ± 410.0 ^b^	1835.6 ± 330.6 ^c^	<0.001	
	**Furan**								
A18	2-Acetylfuran	1489.8	929.479	1.1132	1897.4 ± 335.0 ^a^	1626.7 ± 113.4 ^b^	1778.7 ± 631.9 ^a^	0.047	fatty, sweet, caramel, nutty, tobacco
A13	2-Acetyl-5-methylfuran	1623.4	1260.667	1.16629	700.2 ± 146.5 ^a^	553.7 ± 76.2 ^b^	740.9 ± 200.1 ^a^	<0.001	nut
A45	2-Pentyl furan	1235.8	488.056	1.254	2734.8 ± 552.9 ^a^	2467.0 ± 627.9 ^ab^	2174.8 ± 1138.2 ^b^	0.033	bean, fruity, earthy, green, vegetable
	**Esters**								
A30	Allyl isothiocyanate-M	1382.5	727.841	1.09708	2224.6 ± 509.5 ^a^	1789.8 ± 317.3 ^b^	1168.6 ± 388.0 ^c^	<0.001	sulfur, pungent, garlic
A31	Allyl isothiocyanate-D	1382	727.025	1.36758	802.9 ± 394.7 ^a^	353.6 ± 186.4 ^b^	201.9 ± 196.2 ^c^	<0.001	
A78	Acetic acid butyl ester-M	1080.8	285.259	1.23975	1187.5 ± 136.8 ^a^	1062.5 ± 112.8 ^b^	629.4 ± 347.9 ^c^	<0.001	fruity
A77	Acetic acid butyl ester-D	1080.2	284.688	1.62402	804.8 ± 349.3 ^a^	460.0 ± 148.3 ^b^	141.8 ± 111.0 ^c^	<0.001	
A104	Butyl propanoate	1148.6	360.367	1.7279	397.2 ± 249.3 ^a^	240.8 ± 71.5 ^b^	223.0 ± 143.2 ^b^	<0.001	earthy, sweet rose
A88	Acetic acid propyl ester	958.3	199.773	1.47595	623.0 ± 170.8 ^a^	351.2 ± 61.7 ^b^	182.8 ± 60.9 ^c^	<0.001	fruity, pear
A87	Methyl propanoate	922.7	181.873	1.32964	642.4 ± 165.9 ^b^	636.7 ± 55.9 ^b^	1541.0 ± 505.2 ^a^	<0.001	fruit, rum
A105	Butyl 2-propenoate	1180.4	403.735	1.69254	421.9 ± 236.4 ^a^	190.8 ± 53.8 ^b^	162.1 ± 94.7 ^b^	<0.001	pungent, fruity
	**Ketones**								
A26	D-Fenchone	1410.5	775.827	1.29762	1482.5 ± 200.8 ^a^	1101.7 ± 159.2 ^b^	876.2 ± 228.1 ^c^	<0.001	
A35	1-Hydroxy-2-propanone-M	1314.8	623.695	1.06882	2376.7 ± 500.7 ^b^	2584.8 ± 277.9 ^b^	3100.3 ± 547.8 ^a^	<0.001	pungent, caramel, fresh
A36	1-Hydroxy-2-propanone-D	1315.2	624.321	1.23408	2912.7 ± 1080.5 ^b^	3798.6 ± 2480.9 ^a^	2493.5 ± 479.2 ^b^	0.007	
A37	2-Butanone, 3-hydroxy-M	1292.5	591.181	1.07027	1147.4 ± 267.3 ^b^	1091.4 ± 255.2 ^b^	1898.0 ± 402.0 ^a^	<0.001	butter, cream
A52	2-Butanone, 3-hydroxy-D	1292.8	591.806	1.33411	867.2 ± 137.5 ^b^	693.9 ± 51.9 ^b^	1209.0 ± 612.9 ^a^	<0.001	
A74	2-Heptanone-D	1187.5	414.183	1.63388	1386.2 ± 415.6 ^a^	1069.9 ± 337.6 ^b^	917.3 ± 553.1 ^b^	<0.001	pear, banana, fruity, slight medicinal fragrance
A99	2-Heptanone-M	1187	413.41	1.26216	1115.5 ± 113.4 ^ab^	1176.8 ± 68.1 ^a^	1083.2 ± 203.7 ^b^	0.036	
A81	1-Penten-3-one	1034.7	248.066	1.31212	3760.1 ± 1135.9 ^a^	2606.2 ± 339.6 ^b^	1217.0 ± 861.9 ^c^	<0.001	strong pungent odors
A83	2-Pentanone	989.3	216.79	1.3678	3361.5 ± 783.3 ^a^	2641.8 ± 589.5 ^b^	1946.6 ± 995.9 ^c^	<0.001	acetone, fresh, sweet fruity, wine
A95	3-Penten-2-one-M	1135.9	344.268	1.07465	160.7 ± 54.4 ^b^	268.5 ± 47.8 ^a^	263.6 ± 39.5 ^a^	<0.001	fruity, turns into spicy during storage
A98	3-Penten-2-one-D	1138.4	347.435	1.34621	5146.3 ± 484.6 ^a^	4336.9 ± 242.5 ^b^	2673.7 ± 890.8 ^c^	<0.001	
A90	2-Propanone	831.1	142.855	1.11774	11380.0 ± 788.2 ^a^	11109.0 ± 518.0 ^a^	10040.0 ± 1425.8 ^b^	<0.001	acetone, fresh, sweet fruity, wine
A86	2-Butanone	912.6	177.101	1.24839	5011.7 ± 897.3 ^a^	4299.3 ± 831.5 ^b^	3314.8 ± 1568.1 ^c^	<0.001	fruity, camphor
A50	1-Octen-3-one	1313.6	621.99	1.68755	1028.1 ± 297.8 ^a^	676.2 ± 209.9 ^b^	381.8 ± 316.0 ^c^	<0.001	strong earthy, mushroom, vegetable, fishy, chicken
A56	2-Octanone	1289.3	584.723	1.76483	257.4 ± 113.9 ^a^	165.5 ± 56.6 ^b^	139.2 ± 48.2 ^b^	<0.001	moldy, ketone, milk, cheese, mushroom
A64	2-Hydroxy-2-methyl-4-pentanone	1369	705.786	1.13888	231.7 ± 31.4 ^b^	187.1 ± 26.1 ^b^	354.4 ± 155.3 ^a^	<0.001	mild, pleasant
A103	4-Methyl-2-pentanone	1013.6	232.746	1.17473	501.6 ± 64.0 ^b^	535.1 ± 96.0 ^b^	781.5 ± 242.5 ^a^	<0.001	ketone
A115	2,3 Butanedione	980.5	211.786	1.17268	325.6 ± 67.6 ^a^	367.8 ± 101.0 ^a^	338.2 ± 62.4 ^a^	0.110	butter, popcorn, sweet taste, sour rice
	**Others**								
A66	Dimethyl trisulfide	1376.2	717.487	1.30222	141.8 ± 48.4 ^b^	137.6 ± 65.4 ^b^	306.8 ± 250.8 ^a^	<0.001	fresh onion, mint, spicy
A67	2,4,5-Trimethylthiazole	1389.7	739.957	1.15652	375.2 ± 137.0 ^a^	230.0 ± 36.8 ^b^	122.1 ± 31.0 ^c^	<0.001	cocoa, chocolate, caramel, nutty
A109	1,2-Dimethylbenzene	1228.7	476.488	1.06399	252.5 ± 50.1 ^a^	279.8 ± 75.5 ^a^	140.3 ± 27.8 ^b^	<0.001	geranium
A108	(+)-Limonene	1202.9	436.658	1.21284	236.8 ± 59.9 ^b^	451.6 ± 99.5 ^a^	223.5 ± 32.7 ^b^	<0.001	lemon, sweet, orange, pine oil
A3	1				1265.5 ± 955.5 ^a^	797.5 ± 120.4 ^b^	916.4 ± 207.9 ^b^	0.006	
A10	2				648.9 ± 155.0 ^b^	664.3 ± 91.7 ^b^	1495.3 ± 535.9 ^a^	<0.001	
A1	3				2306.4 ± 440.9 ^a^	2437.1 ± 265.8 ^a^	2153.8 ± 872.4 ^a^	0.178	
A44	4				4517.7 ± 510.3 ^a^	3394.4 ± 454.0 ^b^	2735.5 ± 1034.8 ^c^	<0.001	
A33	5				3059.3 ± 311.8 ^a^	2951.0 ± 133.8 ^a^	2306.8 ± 455.2 ^b^	<0.001	
A34	6				3197.1 ± 890.5 ^a^	1972.7 ± 345.8 ^b^	1222.2 ± 669.1 ^c^	<0.001	
A40	7				859.7 ± 167.3 ^a^	614.2 ± 83.7 ^b^	357.3 ± 155.4 ^c^	<0.001	
A48	8				1113.8 ± 247.8 ^a^	884.4 ± 123.4 ^b^	448.4 ± 176.4 ^c^	<0.001	
A51	9				694.9 ± 129.0 ^a^	498.8 ± 88.4 ^b^	344.5 ± 153.5 ^c^	<0.001	
A54	10				825.4 ± 401.4 ^a^	380.7 ± 95.6 ^b^	267.3 ± 76.6 ^b^	<0.001	
A58	11				1300.1 ± 524.9 ^a^	648.4 ± 136.5 ^b^	493.9 ± 156.0 ^b^	<0.001	
A62	12				472.5 ± 27.4 ^a^	459.9 ± 97.4 ^a^	458.2 ± 132.5 ^a^	0.820	
A61	13				394.1 ± 89.1 ^b^	291.7 ± 46.1 ^b^	850.8 ± 608.7 ^a^	<0.001	
A68	14				126.0 ± 22.5 ^b^	122.4 ± 16.0 ^b^	218.4 ± 125.8 ^a^	<0.001	
A84	15				2092.8 ± 277.4 ^b^	2468.1 ± 109.6 ^a^	2074.8 ± 218.7 ^b^	<0.001	
A102	16				251.9 ± 39.6 ^c^	358.6 ± 29.2 ^b^	434.1 ± 114.8 ^a^	<0.001	
A89	17				1392.4 ± 120.9 ^a^	1366.5 ± 72.2 ^a^	1159.4 ± 326.2 ^b^	<0.001	
A106	18				1105.1 ± 316.6 ^a^	783.6 ± 147.0 ^b^	532.8 ± 220.8 ^c^	<0.001	
A114	19				446.1 ± 84.7 ^b^	367.7 ± 36.4 ^c^	521.9 ± 85.8 ^a^	<0.001	

Note: RI represents relative retention index, Rt represents retention time, and Dt represents relative migration time. Signal intensities were shown as mean ± standard deviation (*n* = 30). Significant differences were determined by Duncan’s test (*p* < 0.05) and indicated by superscript letters. Odor description obtained from https://www.thegoodscentscompany.com/index.html (accessed on 20 June 2024).

## Data Availability

The original contributions presented in the study are included in the article. Further inquiries can be directed to the corresponding author.
